# Is traditional marketing mix still suitable for hotel banquets? An empirical study of banquet marketing in five-star hotels

**DOI:** 10.3389/fpsyg.2022.973904

**Published:** 2022-09-02

**Authors:** Jie Yin, Sha Fang, Yun Cheng

**Affiliations:** ^1^College of Tourism, Huaqiao University, Quanzhou, China; ^2^College of Management, Shenzhen Polytechnic, Shenzhen, China

**Keywords:** five-star hotels, banquet marketing, marketing effect, signaling theory, marketing mix strategies

## Abstract

Are traditional marketing mix strategies still suitable for hotel banquet marketing? Using the binary logistic regression analysis method, this study used 763 banquet sales records at the Quanzhou Hilton Hotel to comprehensively test the influence of traditional marketing mix strategies on banquet marketing effects. By focusing on new marketing methods (such as video, the Internet, and WeChat marketing), this study tested the effectiveness of traditional marketing strategies in the new media era. The findings revealed that a combination of products is easier to market than a single product, whereas price is still a key factor in hotel banquet marketing. However, sales channels and personal identity have no significant effects on banquet marketing. Finally, based on the failure cases analysis, this study proposed a feasible path for promoting banquet marketing.

## Introduction

“Banquet” has been understood as a socially necessary form of diet, also called a party, feast, or wine party (Zhou, 2017). With the development of social structures, hotel banquets tend to have various expressions (Lau and Hui, 2010), such as wedding ceremonies, company dinners, meetings, private banquets, large gatherings, product demonstrations, small theatrical performances, reports, press releases, and dances (Dev and Olsen, 2000; Soh, 2009; Lau and Hui, 2010; Wang et al., 2019). Hotel banquet is an essential source of profit in five-star hotels (Zhou, 2017). In China, the banquet departments of five-star hotels typically cooperate with the government, especially in middle and small cities. However, five-star hotels have recently faced a serious decline in revenues due to government policy changes and cannot obtain stable income anymore based on government reservations. Thus, the hotel banquet departments have shifted their focus to enterprise, individual consumption, and effective marketing tools to attract the target market have become an urgent need.

“Marketing” refers to the process of social management in which individuals or groups benefit from creating or exchanging products and values (Kotler et al., 2011). Successful marketing can improve hotel occupancy and repurchase rates to increase corporate earnings (Jeffrey and Hubbard, 1994). In 1953, Neil Borden proposed the marketing mix concept, formally established in the book “Concept of Marketing Mix” in 1964 (Borden, 1964). After that, McCarthy proposed the 4PS marketing mix strategy (Mccarthy, 1984), which includes four elements: Product, Price, Place, and Promotion. It has played a leading role in marketing and business research for a long time. With the development of the service industry, service marketers found that the traditional 4PS marketing mix model no longer met their needs (Helm and Gritsch, 2014). Accordingly, the marketing mix strategy was developed from 4PS to 5PS (adding people) (Judd, 1987), 7PS (adding people, physical evidence, and process management) (Philip and Gary, 1989), and 11PS (adding people, public relations, probe, partition, prioritization, power, and position) (Bradford and Boyd, 2020).

Hotel marketing has always been a key topic in academic circles (Muala, 2012; Jung et al., 2013; Leonidou et al., 2013; Saniba et al., 2013). In recent years, new marketing methods have been widely used in hotel marketing. Lim et al. (2019) emphasized that hotel marketers need to recognize the importance of media elements in hotel marketing, identify, attract, and communicate with customers at different consumption stages, and predict the development trend of future marketing activities. Thus, hotel managers ought to track the feedback and evaluation of consumers on the website timely to make a corresponding managerial strategy. Furthermore, hotel marketing should adopt a digital competitive strategy (De Pelsmacker et al., 2018). Additionally, personalized needs are also an important aspect of hotel marketing concerns, especially for high-leisure sports groups (Yang et al., 2018).

With the development and application of new media and marketing methods in hotel marketing, is the traditional marketing strategy mix still fit for hotel banquet marketing? To our knowledge, there is no empirical testing and discussion on the effects of specific marketing methods on hotel marketing. Therefore, this study aimed to use the signaling theory to explore the application of traditional marketing strategies to the effect of hotel banquet marketing.

This study contributed to the existing literature as follows. First, it could be the first time to put the traditional marketing mix strategy into hotel banquet marketing and explore its differential influence. Second, by focusing on new marketing methods (such as video, the Internet, and WeChat marketing), this study tested the effectiveness of traditional marketing strategies in the new media era. Third, this study summarized the failure factors of hotel banquet marketing and provided effective management strategies for it. Fourth, this study employed signaling theory to test the effects of the traditional marketing mix on banquets marketing, thereby broadening the application of signaling theory.

## Literature review and hypothesis

### Signaling theory

Signaling theory is applied to solve the information asymmetry between two parties due to differences in knowledge in the organization and business environment. Some management scholars increasingly rely on signaling theory to explain phenomena like stakeholder decisions and behaviors (Taj Saud, 2016; Seong et al., 2018). There are three essential parts to signaling theory: the signal sender, signal receiver, and signal itself. Signal senders are always insiders, such as executives or managers, armed with a mass of information, including organizations and products (Connelly et al., 2010). In addition, signal receivers are outsiders who lack information about products and organizations but would like to receive the information. The last one is the signal itself. Generally speaking, insiders must decide whether to communicate this information to outsiders. The signal sender and signal receiver will reduce information asymmetry through signal transmission and selection (Connelly et al., 2010), and the signal is information released by one party to influence the outcome of the other party’s decision (Soh, 2009).

Signal theory has been used in tourism (Smith and Font, 2014; Su et al., 2018). Smith and Font (2014) explored how volunteer tourism operators communicated responsibility and established the relationship between responsibility and price signaling using signaling theory. In the process of hotel marketing, hotel marketers send signals to consumers. Consumers judge the information quality and then decide whether to buy a hotel banquet product after accepting the signal. The signals with effective quality can encourage consumers to have a positive perception and achieve the purpose of product purchasing. On the contrary, signals with negative quality may result in consumers negatively perceiving products and marketing failure. Therefore, we selected signaling theory to explore the effectiveness of traditional marketing strategies for banquets.

### Hypotheses

#### Product

After investigating the negative information on the hotel website platform, Loo and Leung (2016) found that 50% of the negative comments were related to hotel products. Berezina et al. (2015) also found that hotel facilities, equipment, and other physical properties accounted for numerous negative comments. Accordingly, the product is critical in customers’ consumption. The product can be divided into single and composite products. Single products are characterized by simplicity and lower prices, whereas composite products are characterized by complexity and higher prices because of multi-service contents.

Signaling theory points out that it is difficult for consumers to deal with information overload because it increases their evaluation cost and even causes anxiety and tension (Heitmann et al., 2007). There are various types of banquet products in five-star hotels, mainly including pure catering, outside catering, meetings, and weddings. Pure catering is a single product, which is mainly related to time, place, price, and menu. Outside catering is also a single food product, which transfers product consumption from the hotel interior to the hotel exterior. Meetings and weddings are product mixes. A meeting is the most complex product among hotel banquets because it is necessary to provide accommodation services, guidance services, conference facilities, and equipment. Furthermore, s wedding banquet is different from meeting banquet products. Newlyweds often spend considerable time comparing different wedding banquet products, focusing on food quality, hall facilities, and wedding room preparation. It is not difficult to establish that the complexity of product categories impacts the effectiveness of banquet marketing. According to signaling theory, the more complex the hotel banquet product category, the more factors consumer should consider. Accordingly, we proposed Hypothesis 1 (H1), as shown below:

**H1: A single product is more likely to be marketed successfully than a product mix.**

#### Price

Price is a significant factor that can influence consumers’ psychology and decisions (David, 1996; Nuseir and Madanat, 2015). A higher price sometimes may not symbolize higher quality, meaning more money must be paid (Kim and Hyun, 2011). Muala (2012) pointed out that the product price is a sensitive factor in the purchase process. When a product is regarded as a low price, it will be attractive to purchase during the selection phase. In addition, price is also a major factor for satisfaction and loyalty; that is, customers will carefully perceive if they are getting the most benefit from purchasing (Nuseir and Madanat, 2015). According to signaling theory, a low price makes consumers perceive the hotel banquet product positively and enhances their purchase willingness. Furthermore, previous research has demonstrated that the lower the hotel price, the more likely customers will stay in the hotel (Wei et al., 1999). We then proposed Hypothesis 2 (H2), as shown below:

**H2: The lower the price of banquet products, the easier it is to sell them successfully.**

#### Promotion

Promotion has been widely used in marketing, which can shorten the process of product sales, encourage consumers to buy and re-buy, and improve performance through aggression and anti-aggression competition. Short-term price reductions can bring immediate benefits, while promotional events with long-term goals could build brand equity (Keller, 2008). In addition, some studies have implied the positive benefits of promotion. Saniba et al. (2013) found that promotion activities can affect consumers’ thinking ways, moods, experiences, and purchasing behavior. Xiong and Hu (2010) also found that promotion effectively attracts potential consumers in the hotel reservations process. Queenmary and Shivany (2019) argue that managers can use price and promotional activities to increase seasonal sales because most consumers are always attracted by discounts, gifts, rebates, and special offers. Studies have also shown that price promotion makes it easier for products to successfully sell (John, 2004). According to signaling theory, promotion activities can make consumers perceive products as having excellent quality and reasonable prices. It also conveys a positive signal to consumers to enhance their willingness to pay. We then proposed Hypothesis 3 (H3), as shown below:

**H3: It is easier for banquet products with price promotion to succeed in marketing.**

#### Place

A place is one of the most important assets of an enterprise, which is a path that can take the product from enterprises to consumers. This path includes sales agencies, agents, distributors, retail stores, and so on (Goeldner and Ritchie, 1980). The research by Jung et al. (2013) showed that the “place” played an important role in the process of hotel banquets, and multiple marketing places could effectively improve the success rate of hotel marketing. It was found that the sales place of high-star hotels is conservative, which is mainly divided into five channels: social inquiry, networks and telephones, friends’ recommendations, internal recommendations, and business investigations of competitors.

Friends’ recommendation is a key to finding useful information on social networks (D’cunha and Patil, 2015), and subjective factors can affect consumers’ purchase decisions (Erawan, 2016). To a certain extent, it would improve the public reputation and win trust when recommended by friends. There are similarities between internal and friends’ recommendations, but it is difficult to establish trust using detection networks and telephone channels for consumers. Thus, it could be proposed that:

**H4: Customers who come because of internal and friend recommendations are more likely to buy banquet products than those based on detection, networks, and telephone channels.**

#### People

As discussed in the introduction section, it is not difficult to find that “people” play an important role in the service industry from the expansion of 4P to 11P. Moreover, relevant studies have also confirmed that “people” represent an essential element in the marketing mix strategy (Gibbs and Ivy, 2007; Kushwaha and Agrawal, 2015).

Customers’ behavior, such as purchasing a product or service, is based on trust (Rodgers and Harris, 2003; Okazaki, 2007), including confidence in products, brands, businesses, and sales staff. Customers will generally not buy products without basic trust (Seo et al., 2017). Customers’ trust in sales staff is one of the important factors affecting customers’ purchase behavior. Yuniawati et al. (2019) suggested that hotel sales staff should pay attention to interacting with consumers to establish a trusting relationship. Previous studies have also confirmed that consumers’ familiarity with and trust in sales staff affects customers’ satisfaction and loyalty (Kukanja et al., 2016).

In the process of hotel banquet marketing, there is multiple sales staff, from consulting banquet services to complete banquet services. However, frequent changes in sales staff will make it difficult for consumers to establish a relationship with sales staff to create a sense of trust and dependence. When the sales staff has not changed from the beginning to the end, it can better recommend a hotel banquet product and provide high-quality service for consumers. According to signaling theory, consumers are more likely to buy products when they receive a good signal of product quality. We then proposed Hypothesis 5 (H5), as shown below:

**H5: Consumers served by a sales staff from consultation and purchase to service are more likely to succeed in marketing than those who follow up with numerous sales staff.**

## Materials and methods

### Data

The Quanzhou Hilton Hotel is located in the largest metropolitan area in Fujian province and in the heart of Quanzhou city. It is close to leisure and entertainment destinations, such as Kaiyuan Temple, Qingyuan Mountain, Wanda Plaza, and Ling show Creative Park. The hotel is well equipped and has 2,940 square meters of meeting room and banquet space, including a 1,368 square meters grand ballroom, a 600 square meters banquet hall, a VIP reception room, a board room, two bridal rooms, and seven meeting rooms, which can be configured for any scale of banquet activities.

We chose the sales records from the Quanzhou Hilton Hotel banquet marketing department as our database. The data recorded 763 banquet sales cases from August 1, 2018, to January 31, 2019, involving 291 successful cases and 472 failed cases. Furthermore, each case recorded 24 pieces of basic information, including case number, product type, case status, time of the first consultation, inquiry channel, consultants, first-time consultant, start time, end time, banquet revenue, participation and presence of promotion, and case notes.

The main objects of this study included six major factors in banquet sales records that are product type (product factor), inquiry channel (place factor), first consultant and follow-up sales (people factor), banquet revenue (price factor), presence of promotion (promotion factor), and whether the marketing is successful.

### Methodology

A binary logistic regression model was employed to explore the influence of the traditional marketing mix on the success of banquets in five-star hotels. The binary logistics regression model is an important method of mathematical analysis, which has been widely used in travel stay time (Jacobsen et al., 2018), tourist behavior (Alén et al., 2016; Mccreary et al., 2019), tourist consumption (Correia et al., 2018), tourist destination flow (Chuang et al., 2020), and tourism enterprise marketing (Romero and Tejada, 2019). The binary logistic regression model emphasizes that dependent variables are dichotomous variables and independent variables can be continuous or non-continuous variables, which can effectively describe the relationship between a dependent variable and multiple independent variables (Raun et al., 2016; Vergori and Arima, 2020). The dependent variable in this study was the marketing result (marketing success or failure). Furthermore, there were five independent variables, namely product type (product factor), inquiry channel (place factor), first consultant and follow-up sales (people factor), banquet revenue (price factor), and the presence of promotion (promotion factor). The specific classification is shown in Table 1. Therefore, this study employed the binary logistics regression model to examine the five key factors affecting the marketing results (marketing success or failure) of hotel banquets.

**TABLE 1:** Classification of variable.

Variable	Variable grading	Successful	Failure	Sample tot	Successful rate
X_1_ product categories	1 = Pure catering	169	340	509	33.2%
	2 = Outside catering	4	4	8	50.0%
	3 = Meeting	100	102	202	49.5%
	4 = Wedding	18	26	44	40.9%
X_2_ product price	1 = ¥ 0–15,000	131	158	289	45.3%
	2 = ¥ 15,000–30,000	64	139	203	31.5%
	3 = ¥ 3,000–45,000	27	56	83	32.5%
	4 = ¥ 45,000∼	69	119	188	36.7%
X_3_ promotion	1 = Yes	71	73	144	49.3%
	0 = No	220	399	619	35.5%
X_4_ sales channel	1 = Sales visit	53	98	151	35.1%
	2 = Internet call	235	367	602	39.0%
	3 = Internal recommendation	1	2	3	33.3%
	4 = Friend recommendation	1	1	2	50.0%
	5 = Detect competitor channels	1	4	5	20.0%
X_5_ identity of sales staff	1 = Consulting and selling from a staff	123	209	332	37.0%
	0 = Consulting and selling from different staff	168	263	431	38.98%
Y marketing results	0 = Failure	291	472	763	38.1%
	1 = Successful				

The basic model is as follows:


$$\text{ln}\,\left(\frac{\text{p}}{1-p}\right)=\beta_{0}+\beta_{1}\,{\text{X}}_{1}+\beta_{2}\,{\text{X}}_{2}+\ldots \,+\mu$$

In the formula above, P is the occurrence probability, and 1-p is the probability of an event not occurring. $\frac{p}{1-p}$ is a ratio of the probability of an event occurring to not occurring, which is called the event occurrence probability ratio. When the independent variable (X1) is binary, the coefficient (β_1_) represents the natural logarithm of the ratio of the occurrence of events (X_1_ is 1) to not occurring (X_1_ is 0). The coefficient (β_1_) is usually taken as the natural exponent [EXP (β_1_)] because of its convenience, and the natural exponent [EXP (β_1_)] represents EXP (β_1_) times that an event occurred (X_1_ is 1) and that an event did not occur (X_1_ is 0).

## Results

### Variable analysis

We obtained 763 valid cases by conducting, integrating, and coding case statistics. We then classified banquet products from the categories (product), sales channels (place), product prices (price), promotion, the identity of sales staff, and marketing results. On this basis, we conducted the frequency of hotel banquet marketing case statistics. The sample profile of the hotel banquet is presented in Table 1.

### Model testing

Before model testing, the model’s applicability and fitting degree need to be tested. The results showed that the model met the requirements of an applicative model (*X*^2^ = 23.747, *P* < 0.001). The results also indicated that the OR value was statistically significant, and the research model was highly applicable. Furthermore, we employed the Hosmer and Lemeshow test model (Fagerland and Hosmer, 2012) to estimate the fitting degree. The results showed that the *P* value was 0.942, which is higher than the minimum test standard of 0.05 (Robertson et al., 2008), indicating that the model fit well.

### Hypothesis test

In this statistical analysis, we used the Enter method to incorporate all the independent variables into the model, and Table 2 lists all the independent variables and their parameters. As shown in the table, a column of significance represents the *P* value of the corresponding variable in the model, and a 95% C.I. of EXP (B) represents the OR value of the corresponding variable and its 95% credibility interval.

**TABLE 2:** Results of hypotheses testing.

	Variable	B	S.E	Wald	df	Sig	Exp (B)	95% credibility interval	
								LLCI	ULCI
Product categories	Pure catering			14.771	3	0.002***			
	Outside catering	0.060	0.377	0.026	1	0.873	1.062	0.507	2.223
	Meeting	0.636	0.814	0.610	1	0.435	1.889	0.383	9.317
	Wedding	0.709	0.390	3.312	1	0.069*	2.032	0.947	4.362
Product price	¥ 0–15,000			10.822	3	0.013**			
	¥ 15,000–30,000	0.335	0.201	2.765	1	0.096*	1.398	0.942	2.074
	¥ 30,000–45,000	−0.270	0.221	1.488	1	0.223	0.763	0.495	1.178
	¥ Above 45,000	−0.200	0.286	0.490	1	0.484	0.818	0.467	1.434
Promotion	Product promotion	−0.673	0.217	9.607	1	0.002***	0.510	0.334	0.781
Consultation channel	Sales visit			2.403	4	0.662			
	Internet call	0.692	1.158	0.357	1	0.550	1.998	0.206	19.349
	Internal promotion	0.932	1.147	0.660	1	0.417	2.540	0.268	24.070
	Friends’ recommendation	0.765	1.683	0.206	1	0.650	2.148	0.079	58.120
	Business investigation of competitors	1.699	1.831	0.861	1	0.353	5.468	0.151	197.710
People	Identity of sales	0.153	0.156	0.954	1	0.329	1.165	0.858	1.582
Constant		−1.195	1.154	1.073	1	0.300	0.303		

***, **, and * are significant at the level of 0.01, 0.05, and 0.1, respectively.

As shown in Table 2, product type (*P* = 0.002 < 0.01), product price (*P* = 0.013 < 0.05), and product promotion (*P* = 0.002 < 0.05) were statistically significant, whereas consultation channel and identity of sales were not. Thus, H4 (place factor) and H5 (people factor) are not supported. We then explored the effects of product type, price, and promotion on marketing results.

From the perspective of hotel banquet product type, the partial regression coefficients of product types were 0.060, 0.636, and 0.709, and the corresponding OR values were 1.602, 1.889, and 2.032, respectively. The results indicated that compared with the product type with low value (pure catering), the probability of marketing success of the product type with high value is 1.062 times (outside catering), 1.889 times (meeting), and 2.032 times, respectively, (wedding) of the pure meal.

From the perspective of hotel banquet product price, the partial regression coefficients of product price were 0.335, −0.270, and −0.200, while the corresponding OR values were 1.398, 0.763, and 0.818, respectively. The results showed that compared with the product with a low price (0–15,000), the probability of marketing success of the product with a high price is 1.398 times (1,500–30,000), 0.763 times (3,000–45,000), and 0.818 times (above 45,000), respectively.

From the perspective of hotel banquet product promotion, the partial regression coefficient of product promotion was −0.673, and the corresponding OR value was 0.510. The result demonstrated that compared with product promotion, the probability of marketing success of non-promotion is 0.510.

The above statistical analysis demonstrated that the probability of marketing success increases with the product type complexity. Regarding product price, the price range of banquet consumption with the highest success rate is 15,000–30,000 China yuan, and the price range of the lowest success is 30,000–45,000 China yuan. This implies no obvious correlation between marketing success and product price. In terms of product promotion, banquet marketing with promotion is more likely to succeed than that without promotion.

### Marketing failure analysis

Based on a total of 472 marketing failure cases, 384 cases were selected to determine the reasons for marketing failure effectively. After reading 384 cases with valid records in detail, we tried to divide the reasons for marketing failure into the following categories. The reasons for marketing failure are presented in Table 3.

**TABLE 3:** Reasons for marketing failure.

Head directory	Second-level directory	Third level directory	Counts	Total
The advantages of main business are insufficient	Compared with the same level of hotel competitive advantage is insufficient	Price disadvantage	12	74
		Other factors	5	
	Dates and sites cannot meet customer needs	Not enough for one day	25	
		Not enough for days	9	
	Product preference	Poor sound insulation	1	
		The high cost of the LED	7	
		Space design	11	
	Hotel product positioning	Political needs cannot be met	4	
The advantage of auxiliary business is insufficient	The demand for housing cannot be met	The requested rooms are overbooked	1	17
		Shortage of rooms	5	
	The demand for dining cannot be met	Low-cost working meals are not available	1	
		The customer is not satisfied with the menu	6	
		The customer is not satisfied with the quality	4	
Sales schedule out of order	Customers were taken away by rival hotels		53	124
	The customers are reluctant to give further information		43	
	Lost contact with customers		28	
Locational factor	Customers choose other hotels in the city	The road is inconvenient and the distance is too long	21	60
		Downtown preference	4	
	Customers choose hotels in other cities (Fuzhou, Xiamen)		35	
Customer budget is not enough	Customers choose hotels with below five stars	Shortage of meeting budget	7	47
		Shortage of catering budget	18	
		Shortage of housing budget	4	
		Shortage of comprehensive budget	18	
Other factors	The activities were put off		24	62
	The activities were canceled		38	

Table 3 illustrates 74 banquet marketing failure cases because of insufficient advantages of the main business, accounting for 19.3%. Furthermore, there were 17 banquet marketing failure cases due to insufficient advantages of the auxiliary business, accounting for 4.4% of the total. There are 60 marketing failure cases due to location factors, accounting for 15.6% of the total. There were 47 marketing failure cases caused by insufficient customer budget, accounting for 12.3%. Furthermore, there were 62 marketing failure cases due to event rescheduling or cancelation, accounting for 16.1% of the total.

On this basis, we then divided the failure factors of hotel banquet marketing into controllable and uncontrollable factors. Controllable factors involve insufficient advantages of main and auxiliary businesses and a sales schedule out of order. Uncontrollable factors include location, insufficient customer budget, and event rescheduling or cancelation. We further subdivided the controllable factors and summarized the causes of marketing failure. The response measures for marketing failure are presented in Table 4.

**TABLE 4:** Response measures for marketing failure in detail.

Controllable factor	Reasons for marketing failure in detail	Response measures
The advantages of main business are insufficient	Prices are disadvantaged compared to comparable hotels	Hotel banquet department needs to obtain banquet quotations and discount information about main competitors and adjust its own product price structure and convey positive quality signal to divert customer sensitivity to price in the process of sales.
	LED outsourcing a third-party company, LED fees cannot be reduced	The hotel should convert LED into hotel fixed assets.
	Dates and sites cannot meet customer needs	Relevant hotel departments should discuss different combination methods of large and small banquet halls and forms of banquet tables to create more possibilities.
	The space design does not meet the needs of special banquets	
	Political demand do not meet the needs and banquets with a strong political atmosphere are facing great competitive pressure	Hotel banquet marketing department should strengthen the cooperative relationship with the government banquet and provide customized services for the government banquet.
The advantage of the auxiliary business is insufficient	The demand for housing cannot be met mainly because of the shortage of rooms	Hotel related departments should challenge the form of room combination and appropriately transform the room type with a low utilization rate into the room type with the main demand for banquets.
	The demand for dining cannot be met. Customized menu has no obvious advantage compared with competition and they do not trust the quality of products	Hotel banquet department ought to prepare multi-price and multi-type menus to improve the quality of dishes and increase competitiveness in different banquet marketing strategies and periods.
Sales schedule out of order	Customers were taken away by rival hotels	Hotel banquet staff timely follows up banquet status. Hotels can increase discounts to enhance competitive advantage for high-yield banquet products and build a friendly communication mode with customers.
	The customers are reluctant to give further information	
	Lost contact with customers	

## Discussion and implication

### Conclusion and discussion

Hotel banquets have become the main source of hotel income, and banquet marketing has become an important topic for hotel management (Lau and Hui, 2010; Guan et al., 2015; Wang et al., 2019). Therefore, this study aimed to explore the key factors determining the effect of hotel banquet marketing. Moreover, we also concluded the failure factors of hotel banquet marketing and provided an effective management strategy for the hotel banquet marketing department. We then revealed several important conclusions as follows.

First, a combination of products is easier to market than a single product. Thus, H1 is not supported. Complex banquet products, including conference, dining, and accommodation, are easier to be successfully marketed than pure catering banquet products. A combination of products has gradually become a way of product marketing with the development of the economy, and a related study demonstrated that changing a product combination can enhance the attractiveness of destinations and improve consumer satisfaction (Myriam, 1991). Furthermore, the price of a combined product is normally lower than that of a single product, which also works for hotel banquet products. The higher the degree of the banquet products combination, the easier it is to attract consumers, which means that consumers can buy the same number of products at a lower price.

Second, the price of the hotel banquet product affects the marketing effect to a certain extent, and H2 is partially supported. The price of banquet products below 30,000 Chinese yuan is easy to sell successfully, whereas the price above 30,000 Chinese yuan is difficult to sell. Chan and Wong (2016) also discovered that price impacts hotel selection for consumers, but it is not the only factor. Other factors, such as service quality, and even convenience, also influence customers’ choices. In addition, some research pointed out that although price implies high quality, it cannot create loyalty to the brand; that is, price is not the only factor in choosing products (Helsen and Schmittlein, 1994). Similarly, consumers do not take price as the decisive factor in the hotel banquet selection but consider a combination of multiple factors. Besides, the failure cases of hotel banquets in this study further support this point of view.

Third, banquet marketing with promotion is easier to succeed at than banquet marketing without promotion, and H3 is supported. It is consistent with the previous research. For example, Saniba et al. (2013) argued that promotional activities would affect customers’ thinking modes, emotions, experiences, and purchasing behavior. Sales promotion has always been an effective means of attracting potential customers (Dawes, 2004; Xiong and Hu, 2010). Queenmary and Shivany (2019) found that consumers tended to be attracted by discounts, gifts, rebates, and preferential prices through the research of promotional activities of retail stores. A Thai tourism demand survey also found that promotion had the most influence on demand (Nonthapot and Thomya, 2020). Therefore, people are always attracted to hotel banquets with sales promotions.

Fourth, a “place” cannot affect the marketing effectiveness of a hotel banquet, and H4 is not supported. Consistent with existing study evidence, this research implies that a place is not a determinant factor for tourists’ demand (Nonthapot and Thomya, 2020). From the perspective of marketing channels, the hotel banquet product, as a product marketed to a specific group, is difficult to change consumers’ purchasing intentions through channel conversion. Therefore, different channels have little effect on banquet marketing results. Furthermore, the identity of the same person has no significant impact on the marketing effectiveness of five-star hotels, thus failing to support H5. We argue that most consumers focus on the banquet product mix, product quality, and price, rather than on sales staff. Therefore, many customers value the quality of service rather than the provider of the service.

Finally, marketing failure reasons were divided into six categories in the process of analyzing marketing failure cases: the advantages of the main business are insufficient, the advantages of the auxiliary business are insufficient, the sales schedule is out of order, locational, customer budget is not enough, and the activities were put off or canceled. This research outcome is in line with related studies in the literature. Ennew and Schoefer (2003) divided service system external failures into three types: unavailable service, unreasonably slow service, and other core service failures. In addition, a budget is an internal precondition for purchasing products, which also determines the marketing result of a hotel banquet.

### Implications

The primary objective of this study was to provide management recommendations for hotel banquet departments, especially for hotel banquet marketing departments. Based on the results of this study, we put forward seven management suggestions for hotel banquet marketing departments as follows.

First, price is the most sensitive factor in banquet marketing. The larger the amount involved in banquet consumption, the more difficult the marketing will be. Therefore, the banquet management department should increase income, reduce expenditure, and then reduce external consumption. On the one hand, the hotel banquet marketing department should plan the price structure of banquet products scientifically and reduce outsourcing projects in banquet activities. On the other hand, hotel banquet marketing departments should comprehensively conduct marketing research to understand the market situation, combine it with their positioning to reinforce the hotel’s features, and maintain competitive advantages with the same level of hotels from the perspective of long-term interests instead of blindly lower pricing products. Furthermore, hotels ought to scientifically plan the price structure of banquet products, hide outsourcing items, and reduce the customers’ sensitivity to their prices in the form of discounts, complimentary hotel services, and rent-free hotel facilities and equipment to prevent customers from being cut off by competitor hotels in the process of booking or comparing banquet products.

Second, the research demonstrated that a combination of products was easier to market than a single product in hotel banquet marketing. That is, consumers can buy more products for less money. Therefore, hotels should conduct market research to understand the preferences of the public for the banquet product and then moderately increase the additional values of hotel banquet products, such as using the stage, setting the scene, and guiding the electronic screen. Besides, hotels also need to create a banquet marketing atmosphere with a sense of ceremony to achieve a surprise effect according to the needs of the comprehensive service. For example, hotels should provide active blessings and additional gifts to make customers feel the service temperature.

Third, the auxiliary business has an essential influence on the success of banquet marketing in five-star hotels. The key to improving the success rate of banquet marketing is ensuring banquet catering and high-quality hotel rooms. Therefore, the hotel banquet department should strengthen contact and cooperation with guest rooms and catering departments to respond to customer needs promptly. More precisely, the banquet department needs to count the number of guests staying on the same day and timely interface with the guest room department to meet consumer demand. In addition, due to the cycle of menu customization in banquet catering, the banquet department needs to maintain close contact with the catering department to update different types of menus, understand the seasonal dishes, and develop a menu customization system to quickly respond to customer demands, propose marketing countermeasures, and reduce unnecessary losses.

Fourth, it is necessary to use banquet space reasonably. Due to the fixed nature of the space, it is bound to be unable to meet all the demands of the banquet. Therefore, banquet departments must employ different combination methods of large and small banquet halls, conference halls, and table settings in banquet marketing. At the same time, hotels should actively develop special expressiveness of outdoor space and non-banquet space to form personalized banquet services, such as high-rise terraces, indoor swimming pools, and presidential suites. The hotel banquet departments also need to adopt movable partitions by semi-automatic or automatic control to divide the space and maximize the space according to the number of people.

Fifth, employees remain an important asset for banquets. Hotels should use marketing personnel to support industrial development and focus on training in the marketing management process. The service quality and effectiveness of the hotel banquet depend on the professional skill level of the hotel banquet staff. Furthermore, the banquet sales staff should directly contact potential customers as public relations representatives and through hotel business cards. Therefore, hotels should strengthen training to improve comprehensive quality and professional skills for banquet sales. At the same time, hotels should pay attention to the needs of employees, cultivate their loyalty, and allow employees to send positive signals to customers through professional, efficient, and high-quality service capabilities.

### Limitations and further research

The study proposed a feasible path of promoting the marketing effects of banquets in five-star hotels with the failure cases and reasons. However, there are still some limitations to the current research. First, the study revealed key factors that affected hotel banquet marketing results using the banquet department’s marketing case at the Hilton Hotel Quanzhou. However, future research should pay more attention to data analysis of diversified, multi-stage, and multi-type hotels to explore the key factors affecting the marketing effects of hotel banquets comprehensively. Additionally, physical evidence and process management are important factors influencing banquet marketing effects that need to be considered in future research. Finally, future studies could explore the failure cases of hotel banquet marketing and the key factors leading to its failure.

## Data availability statement

The original contributions presented in this study are included in the article/supplementary material, further inquiries can be directed to the corresponding author.

## Author contributions

JY: conceptual framework. SF: writing and editing. YC: data collection and data analysis. All authors contributed to the article and approved the submitted version.
